# Highly diverse and antimicrobial susceptible *Escherichia coli* display a naïve bacterial population in fruit bats from the Republic of Congo

**DOI:** 10.1371/journal.pone.0178146

**Published:** 2017-07-12

**Authors:** Kathrin Nowak, Jakob Fahr, Natalie Weber, Antina Lübke-Becker, Torsten Semmler, Sabrina Weiss, Jean-Vivien Mombouli, Lothar H. Wieler, Sebastian Guenther, Fabian H. Leendertz, Christa Ewers

**Affiliations:** 1 Epidemiology of highly pathogenic microorganisms, Robert Koch Institute, Berlin, Germany; 2 Department of Migration and Immuno-Ecology, Vogelwarte Radolfzell, Max Planck Institute for Ornithology, Radolfzell, Germany; 3 Zoological Institute, TU Braunschweig, Braunschweig, Germany; 4 Institute of Evolutionary Ecology and Conservation Genomics, Universität Ulm, Ulm, Germany; 5 Institute of Microbiology and Epizootics, Freie Universität Berlin, Berlin, Germany; 6 Microbial Genomics, Robert Koch Institute, Berlin, Germany; 7 Laboratoire nationale de la santé publique, Brazzaville, Republic of Congo; 8 Robert Koch Institute, Berlin, Germany; 9 Veterinary Faculty, Institute of Hygiene and Infectious Diseases of Animals, Justus-Liebig-Universität Giessen, Giessen, Germany; Universidade de Aveiro, PORTUGAL

## Abstract

Bats are suspected to be a reservoir of several bacterial and viral pathogens relevant to animal and human health, but studies on *Escherichia coli* in these animals are sparse. We investigated the presence of *E*. *coli* in tissue samples (liver, lung and intestines) collected from 50 fruit bats of five different species (*Eidolon helvum*, *Epomops franqueti*, *Hypsignathus monstrosus*, *Myonycteris torquata*, *Rousettus aegyptiacus)* of two different areas in the Republic of Congo between 2009 and 2010. To assess *E*. *coli* pathotypes and phylogenetic relationships, we determined the presence of 59 virulence associated genes and multilocus sequence types (STs). Isolates were further tested for their susceptibility to several antimicrobial substances by agar disk diffusion test and for the presence of an Extended-Spectrum Beta-Lactamase phenotype. *E*. *coli* was detected in 60% of the bats analysed. The diversity of *E*. *coli* strains was very high, with 37 different STs within 40 isolates. Occasionally, we detected sequence types (e.g. ST69, ST127, and ST131) and pathotypes (e.g. ExPEC, EPEC and atypical EPEC), which are known pathogens in human and/or animal infections. Although the majority of strains were assigned to phylogenetic group B2 (46.2%), which is linked with the ExPEC pathovar, occurrence of virulence-associated genes in these strains were unexpectedly low. Due to this, and as only few of the *E*. *coli* isolates showed intermediate resistance to certain antimicrobial substances, we assume a rather naïve *E*. *coli* population, lacking contact to humans or domestic animals. Future studies featuring in depth comparative whole genome sequence analyses will provide insights into the microevolution of this interesting strain collection.

## Introduction

Bats (Chiroptera) are the second most diverse order of mammals with more than 1300 species and an almost global distribution [1]. Some special features of their morphology, physiology and behaviour make bats unique in their role as reservoirs and distributors for pathogens; above all is the ability of active flight, which enables the spread of pathogens over long distances [2,3]. Over the last decades, bats have been suggested or identified as reservoir hosts for several viruses of considerable human and animal health concern, such as Marburgvirus, Hendra, Nipah or SARS viruses [4–6]. Previous studies have also shown that bats might be sources for bacterial pathogens such as *Leptospira* spp. [7], *Salmonella* spp. [8], *Yersinia* spp. [9] or *Bartonella* spp. [10].

Bats also carry *Escherichia coli* [11–13], a classical component of the intestinal microbiota of humans and most warm blooded animals [14]. Most investigations were restricted to classical microbiological methods [11,12,15,16], whereas further determination of the pathogenic potential of isolated strains has only rarely been performed and was mainly restricted to phylogenetic assignment or detection of intestinal pathogenic *E*. *coli* [13,17–19].

Besides commensal strains, the species *E*. *coli* comprises several zoonotic pathovars causing intra- and extraintestinal diseases in humans and animals, such as diarrhoea, septicaemia, urinary tract infections or meningitis [14,20]. While much is known about the prevalence and pathogenic potential of *E*. *coli* in humans and domestic animals, little is known about *E*. *coli* in wildlife. Few studies reveal mainly commensal strains in captive wild animals [21,22], whereas others reported zoonotic and potentially extra intestinal pathogenic *E*. *coli* (ExPEC) strains originating from wildlife, indicating that wildlife might serve as a source or reservoir of virulent *E*. *coli* strains [13,23–25]. However, to which extent pathogenic *E*. *coli* from bats might be of zoonotic relevance is difficult to assess, as data on the phylogenetic types, e.g. determined by multilocus sequence typing (MLST), are not available. Food represents one of the main infection sources for intestinal pathogenic *E*. *coli* and there is also scientific support for foodborne infections with ExPEC [26,27]. As bats provide a considerable protein source for people in many African countries [28–30], it would be highly desirable to get more insight into the prevalence and genetic background of *E*. *coli* from these animals. Another important aspect is that wild animals have increasingly been recognized as carriers and putative distributors of antimicrobial resistant (AMR) *E*. *coli* [31–35]. Indeed, bats have been shown to harbour AMR *E*. *coli*, demonstrating that they can be considered an important pool for genetic material of bacteria [17,23,36,37].

The objective of this study was to determine the presence of pathogenic *E*. *coli* in free-ranging fruit bats from the Republic of Congo by investigating virulence gene profiles, phylogenetic types and antimicrobial resistance patterns of the strains.

## Materials and methods

### Animal sampling

This study was performed after written authorization by the Ministry of Scientific Research (Délégation Général à la Recherche Scientifique et Technologique) and the Ministry of Forest Economy and Wildlife of the government of the Republic of Congo.

**Fig 1:**
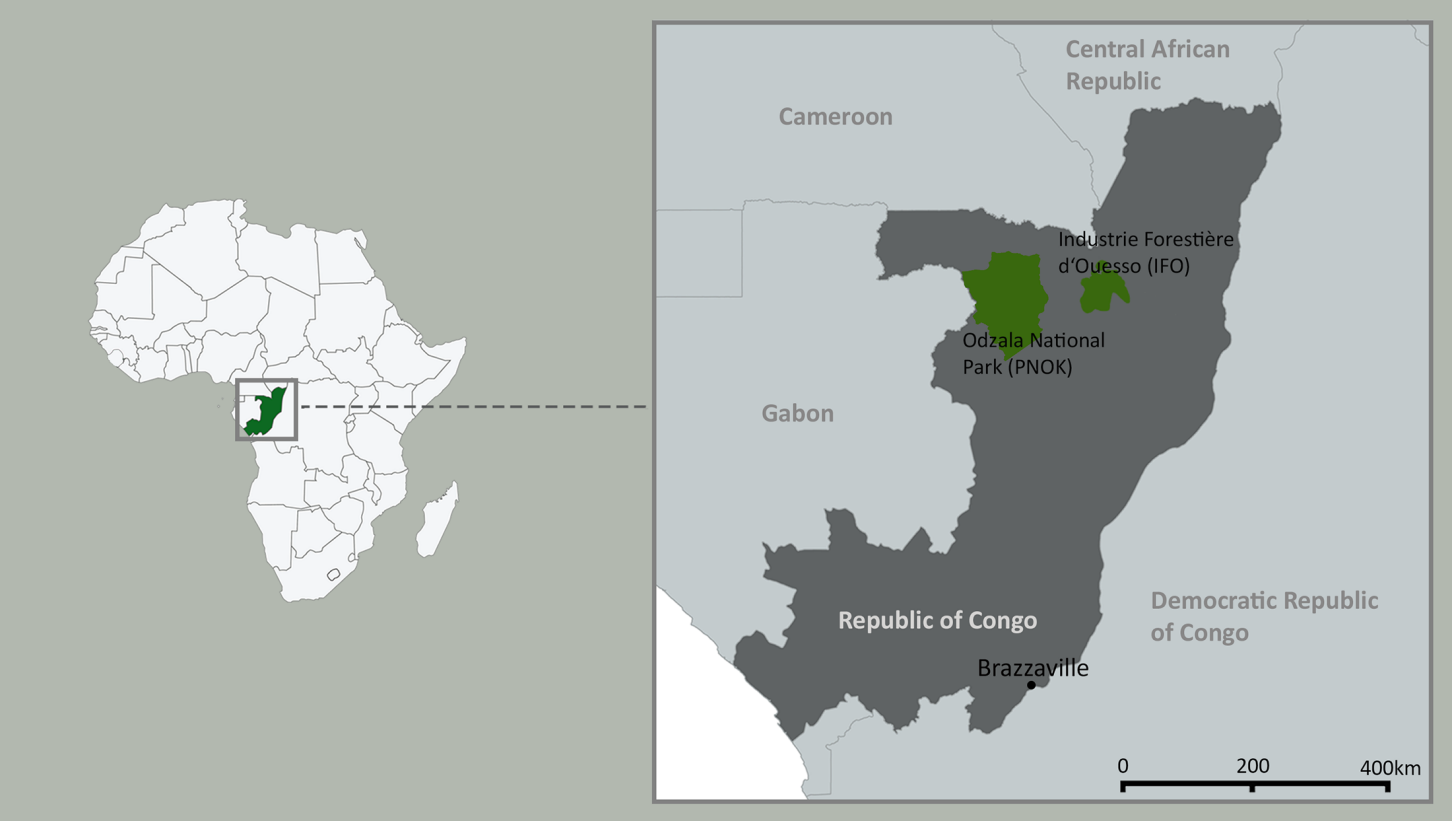
Sampling sites of African bats in the Republic of Congo. Parc National d’Odzala-Kokoua (PNOK); Industrie forestière d’Ouesso (IFO).

Within the context of a larger study on ecology and infectious diseases of African bats [38,39], 50 fruit bats comprising five species (*Eidolon helvum* n = 2; *Epomops franqueti* n = 3; *Hypsignathus monstrosus* n = 2; *Myonycteris torquata* n = 40; *Rousettus aegyptiacus* n = 3) were screened for *E*. *coli*. None of the aforementioned bat species is regarded as threatened according to the IUCN redlist (http://www.iucnredlist.org). Animals were caught with mist nets during two field sessions in 2009 and 2010 in two different areas in the Republic of Congo (RC) (Fig 1). Thirty-two bats were sampled in the logging concession Industrie Forestière d’Ouesso (IFO) in the north of the RC (UTM 33 N 597017 166972); another 18 bats came from Odzala National Park (Parc National d’Odzala-Kokoua, PNOK, UTM 33M 487319 9934847) in the north-western part of the RC almost 200 km west of IFO. All animals appeared healthy at the time of sampling.

Bats were anesthetized in the field with Rompun 2% (Xylazin 20mg/ml) and Ketamin 10% (100mg/ml) [40]. Animals were euthanized by bleeding them with cardiac puncture. Tissue samples of spleen, liver, kidney, lung and intestines were aseptically taken. Intestines were removed last to avoid contamination of organs with intestinal content. All samples were immediately preserved in liquid nitrogen and stored at -80°C until further analyses.

### Cultivation of organ tissues

Sixty-four frozen tissue samples from 50 animals (intestines (with fecal content) (n = 46), liver (n = 6), lung (n = 9), and kidney (n = 3)) were used to attempt cultivation of *E*. *coli*. To compensate for sub-lethal injuries of the bacteria due to the freezing process of organs at -80°C, all samples were initially incubated over night at 37°C in brain heart infusion (BHI) broth (Oxoid, Germany). BHI broth was streaked on Columbia blood agar (5% blood), Gassner agar and Chrom orientation agar (Oxoid, Germany) and incubated over night at 37°C. Purple colonies from Chrom orientation agar were confirmed as *E*. *coli* by conventional biochemical tests as described previously [24]. Ability of haemolysis of corresponding colonies was assessed on blood agar. Where biochemical tests revealed ambiguous results, bacterial species were identified with the Api 20E test system (Biomérieux, Germany). One *E*. *coli* isolate per sample was picked and used for further analyses, except in cases *E*. *coli* colonies showed two various morphologies on Gassner agar. Then two *E*. *coli* isolates per sample were taken. All isolates were stored at -80°C in BHI broth with 10% glycerol until further use.

### DNA preparation

Bacterial DNA of the *E*. *coli* isolates was extracted by using the Master PureTM Genomic DNA- Purification Kit for blood version II (Biozym Diagnostic GmbH, Germany) according to the manufacturer’s instruction. DNA was diluted to a working concentration of 50ng/μl.

### Multilocus sequence typing

Multilocus sequence typing (MLST) was carried out as described previously [41,42]. Gene amplification and sequencing were performed by using primers specified on the *E*. *coli* MLST website (http://mlst.warwick.ac.uk/mlst). Sequences were analysed by the software package RidomSeqSphere 0.9.19 (http://www3.ridom.de/seqsphere) and STs were computed automatically.

### Pulsed-field gel electrophoresis

Pulsed-field gel electrophoresis (PFGE) was performed according to a published protocol [43] using restriction endonuclease XbaI. PFGE was applied to reveal the clonal relatedness of *E*. *coli* isolates assigned to the same multilocus sequence types (ST) but isolated from several individuals or from several organs within a single animal. Macrorestriction profiles were compared with the unweighted-pair group method using the average linkage method, and Dice similarity indices were calculated (complete linkage; optimization, 1%; position tolerance, 1.5%), using the BioNumerics software (version 6.6; Applied Maths, Belgium). Copy strains were excluded from further analyses.

### Antimicrobial resistance

Susceptibility towards antimicrobial substances was tested by agar disc diffusion test (ADD), an approved norm for antimicrobial testing of *E*. *coli* according to the standards of the Clinical and Laboratory Standards Institute (CLSI) [44,45]. Other techniques as the determination of the minimum inhibitory concentration might have resulted in a quantitative more accurate statement, however the general interpretation of data would have been identical in the different test techniques wherefore they were not performed in this study.

Bacterial material was suspended in Mueller-Hinton broth II and adjusted to a density of McFarland 0.5. The bouillon was then plated on the surface of Mueller-Hinton II agar and antimicrobial discs containing amikacin (30 μg), amoxicillin/clavulanic acid (20/10 μg), ampicillin (10 μg), cefalexin (30 μg), cefazolin (30 μg), cefovecin (30 μg), chloramphenicol (30 μg), doxycycline (30 μg), enrofloxacin (5 μg), gentamicin (10 μg), marbofloxacin (5 μg), sulfamethoxazole/trimethoprim (23.75/1.25 μg) and tetracycline (30 μg) (BD, Heidelberg, Germany) were added. Plates were incubated over night at 37°C and the isolates were classified as susceptible, intermediate or resistant according to the breakpoints defined by the CLSI [44,45]. *E*. *coli* isolates were additionally tested for phenotypic Extended-Spectrum Beta-Lactamase (ESBL)-production with the ESBL confirmatory test recommended by the CLSI, using cefotaxim (30 μg), cefotaxim/clavulanic acid (30 μg/10 μg), ceftazidim (30 μg) and ceftazidim/clavulanic acid (30 μg/10 μg) [45].

### Virulence genotyping

*E*. *coli* isolates were investigated by PCR for 59 genes coding for 62 virulence factors associated with intestinal and extraintestinal pathogenic *E*. *coli*.

In detail, we targeted genes coding for adhesins (*afa/draBC*, *bfp*, *bmaE*, *csgA*, *ea-I*, *eae*, *fimC*, *focG*, *gafD*, *hra*, *iha*, *mat*, *nfaE*, *papAH*, *papC*, *papEF*, *papGII/III*, *sfa/foc*, *sfaS*, *tsh*), iron acquisition systems (*chuA*, *eitA*, *eitC*, *feoB*, *fyuA*, *ireA*, *iroN*, *irp2*, *iucD*, *iutA*, *sitA*, *sitD* [chromosomal], *sitD* [episomal]), serum resistance (*iss*, *kpsMTII*, *neuC*, *ompA*, *traT*), toxins (*astA*, *cnf1/2*, *hlyA*, *hlyC*, *hlyF*, *sat*, *stx1*, *stx2*, *vat*), invasins (*ibeA*, *gimB*, *tia*) and miscellaneous factors (*cvaC*, *cvi/cva*, *escV*, *etsB*, *etsC*, *malX*, *ompT*, *pic*, *pks*, *puvA*). Primers used in this study were obtained from Sigma Genosys (Steinheim, Germany) and were used in multiplex and single PCR approaches. Primer sequences, including sequences that have been described previously, and coordinates are shown in S1 Table in the supplemental material. Additionally, we tested for the presence of genes encoding for enterotoxins (*est-Ia*, *est-II*, *eltB-I*) and fimbriae (*fanA*, *fedA*, *fasA*, *faeG*, *fim41A*) [46].

The 10 strains that were initially identified as copy strains by MLST and PFGE analysis also shared identical virulence gene profiles and were excluded from subsequent analyses to avoid any bias in the interpretation of data.

### Phylyogenetic grouping

To define phylogenetic groups, we analysed the population structure of *E*. *coli* by applying a Bayesian approach to estimate global ancestry by sampling from the posterior distribution over global ancestry parameters on the sequences of the seven gene fragments used for MLST with STRUCTURE [47,48]. This resulted in the partition of six distinct groups. According to Wirth et al. (2006), the groups were assigned to four main phylogenetic groups A, B1, B2, and D and two hybrid groups AxB1 and ABD [41]. eBURSTV2 analysis (http://eburst.mlst.net/9.asp) was performed to identify clonal complexes (CCs), defined as groups of two or more independent isolates sharing identical alleles at six or more loci.

To reveal the phylogenetic relatedness of the STs identified in the present study to known phylogenetic lineages, we compared them to known representatives of ExPEC (ST62, ST73, ST95) and enteropathogenic *E*. *coli* (EPEC) /atypical EPEC (aEPEC)(e.g. ST15, ST20, ST28, and ST29) (S1 Fig) (http://mlst.warwick.ac.uk/mlst/dbs/Ecoli; last access 10.02.2016). To sort the position of the *Escherichia albertii* strain ST3227, known members of the *Escherichia* second population (isolates Z205 from a parrot [ST125], RL325/96 from a dog [ST133] and E10083 [ST546] from a human) [41,49] and of species *E*. *albertii* were included in the alignment for comparative purposes.

Due to the high relevance of ST131, whole genome sequencing (WGS) was performed on one ST131 strain from a bat recovered in this study. DNA was sequenced using an Illumina MiSeq using 300 bp paired end reads and 100-fold coverage. To perform sequence analyses for phylogenetic relationship, sequence data was assembled de novo using CLC Genomics Workbench v.8 (Quiagen). Sequences obtained were compared to published full genomes [50] and the phylogenetic tree generated with RAxML 8.1.14 [51].

All new assigned STs are available from the MLST website (http://mlst.warwick.ac.uk/mlst/dbs/Ecoli/Downloads_HTML). WGS of the ST131 is available from ncbi (accession number LYRV00000000).

## Results

### Detection rate of *E*. *coli* in bat samples

Overall, 49 *E*. *coli* and one *E*. *albertii* isolate were detected in organ tissues from 30 of 50 tested animals (Table 1). In general, only one *Escherichia* spp. colony per organ sample was further processed, except for cases where morphological differences could be observed on Gassner Agar. MLST analysis of the 50 isolates and subsequent PFGE analysis of isolates from different organs of the same animals revealed that ten out of 50 isolates were likely copy strains (Table 1, selected profiles shown in S2 Fig).

**Table 1:** Occurrence of *E*. *coli* in different African bat species.

Bat species	Nr. *E*. *coli* positives (n)/ animals tested (n)	*E*. *coli* strains (n)/ isolates (n)
*Eidolon helvum*	2/ 3	3/ 4
*Epomops franqueti*	2/ 3	2/ 2
*Hypsignathus monstrosus*	2/ 2	2/ 2
*Myonycteris torquata*	22/ 40	31/ 40*
*Rousettus aegyptiacus*	2/ 3	2/ 2
**Total**	**30/ 50**	**40/ 50**

*One isolate was identified as *E*. *albertii*

### Virulence gene typing and *E*. *coli* pathotype grouping

**Fig 2:**
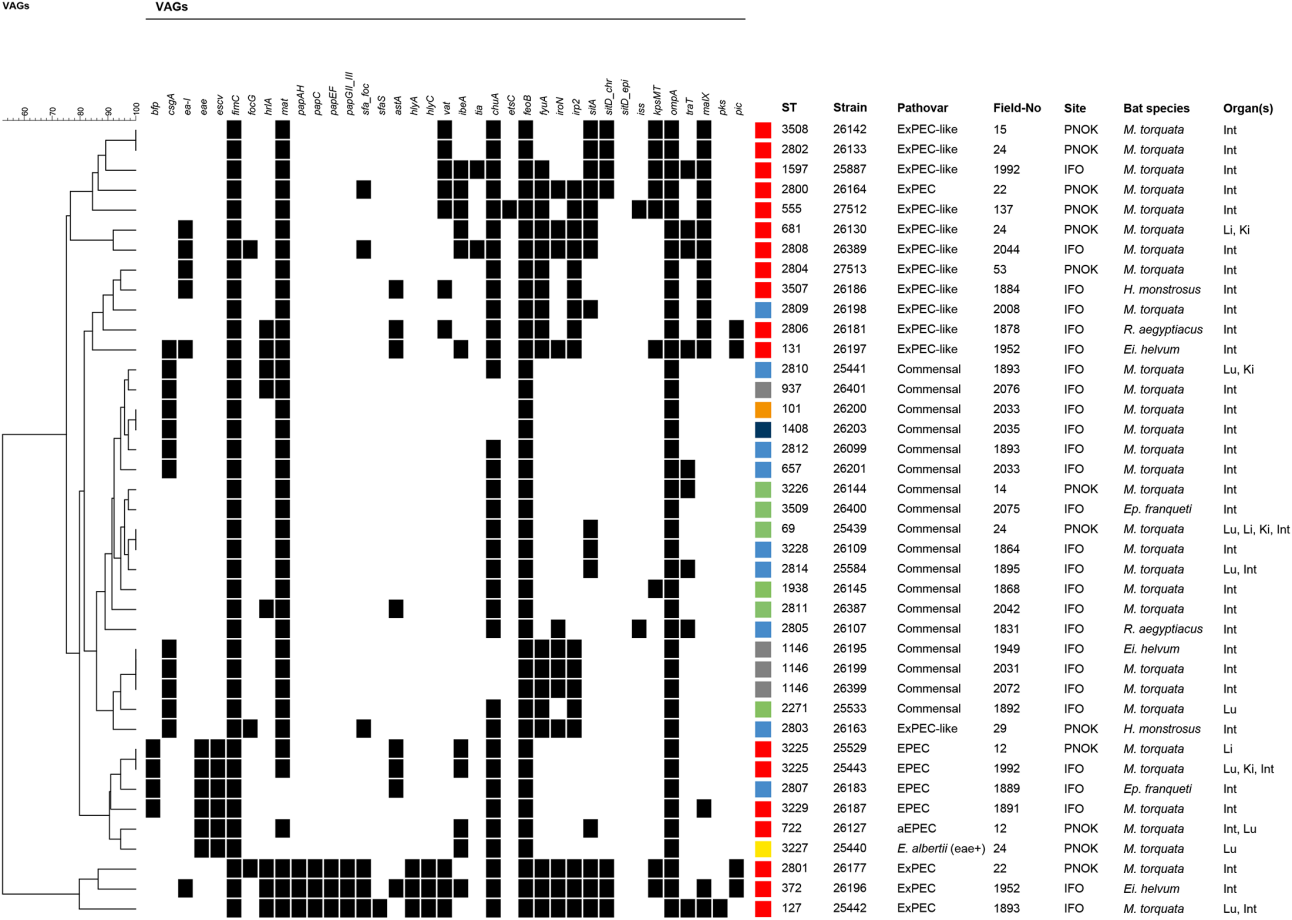
Virulence gene pattern and characteristic features of 39 *E*. *coli* and one *E*. *albertii* strain. Strains of the EcoR group B2 are indicated in red, D in green, B1 in orange, A in dark blue, AxB1 in grey and ABD in light blue; genes absent among strains (*afa/dra*, *bmaE*, *gafD*, *iha*, *nfaE*, *tsh*, *eitA*, *eitC*, *ireA*, *iucD*, *iutA*, *sitD epi*, *neuC*, *ompT*, *cnf1/2*, *hlyF*, *sat*, *stx1*, *stx2*, *cvaA*, *cvi/cva*, *etsB*, *gimB*, *puvA)* are not shown. Abbreviations: ST = sequence type; STC = ST complex; In = intestines; Li = liver; Lu = lung; Ki = kidney; M = *Myonycteris*; Ei = *Eidolon*; Ep = *Epomops;* H = *Hypsignathus*; R = *Rousettus*; RC = Republic of Congo; PNOK = Park National d’Odzala Kokoua (Odzala National Park); IFO = Industrie Forestière d’Ouesso. ** Newly assigned STs are indicated by a diamond.

Shiga-toxin genes *stx1* and *stx2*, which would be predictive of the presence of Shiga-toxin producing *E*. *coli* (STEC), were not detected in any of the 40 tested *Escherichia* spp. isolates. Four isolates (IMT25529, IMT25433, IMT26183 and IMT26187) could be assigned to the EPEC pathovar. These isolates harboured the typical EPEC genes *eae*, *escV*, and *bfp*, which encode the adhesin intimin, a type III secretion system encoded on the locus of enterocyte effacement (LEE), and the plasmid-located bundle forming pili, respectively (Fig 2). One *E*. *coli* isolate (IMT26127) was further identified as aEPEC as it was *bfp*-negative but possessed the *eae* and *escV* genes [52]. Also the *E*. *albertii* isolate (IMT25440), which was obtained from the lung of a *M*. *torquata* from PNOK, was tested *eae*+ and *bfp/stx*-. From one *M*. *torquata* from PNOK with the field no. 12, we could cultivate both a typical EPEC (IMT25529) from the liver and an atypical EPEC from the intestine (IMT26127) and lung (copy strain, no. not listed).

The remaining isolates were sub-grouped into ExPEC, ExPEC-like and commensals (Fig 2). According to a molecular definition previously suggested by Johnson et al. [53], we defined *E*. *coli* isolates as ExPEC based on the presence of ≥2 virulence associated genes (VAGs) including P fimbrial genes *papA* and *papC*, S frimbriae genes *sfa/foc*, afimbrial adhesion genes *afa/dra*, group 2 polysaccharide capsule gene *kpsMTII* and iron acquisition gene *iutA*. However, many VAGs were only discovered after the “ExPEC-definition” had been suggested in 2003 or were found to be of relevance in ExPEC infections after this time point [54–60]. We used a second scheme to categorize the remaining strains based on the presence of at least five ExPEC related genes and termed them “ExPEC-like”. Of note, this categorization is just for practical reasons and does not suggest anything about the strains virulence. Genes included encode for groups II and K1 capsule (*kpsMTII*, *neuC*) adhesins (*ea-I*, *hrA*, *sfa/foc*, *tsh*), toxins (*cnf*, *hlyA*, *hlyC*, *hlyF*, *vat*, and *sat*), invasins (*ibeA*), iron acquisition systems (*chuA*, *sitA*, *sitD*, *etsA/etsB*, *eitA/eitB*, *fyuA*, *ireA*, *iroN*, *irp-2*, *iucD*), a pathogenicity island marker (*malX*), ColV plasmid (*cvi/cvaC*), protectins (*iss*, *ompT*) and a polyketide synthetase (*pks*) (S2 Table). Isolates not fulfilling any of the above criteria were termed commensals. Following these definitions, we identified four ExPEC strains from different organs of three bats. Another 12 isolates fulfilled the criteria of ExPEC-like strains, while 18 isolates were termed commensals.

Irrespective of their pathovar designation, almost all *E*. *coli* isolates carried adhesion-related genes *fimC* and *mat*, outer membrane protein gene *ompA* and iron acquisition gene *feoB* (92.5–100%) (Table 2).

**Table 2:** Distribution of virulence associated genes among *E*. *coli* from African fruit bats.

Category/gene	Positive strains %				
	EPEC / aEPEC (n = 5)	ExPEC (n = 4)	ExPEC-like (n = 12)	Commensals (n = 18)	Total (n = 39)
**Adhesins**					
*afa/dra*	0	0	0	0	0
*bmaE*	0	0	0	0	0
*bfp*	80.0	0	0	0	10.3
*csgA*	0	0	16.7	55.6	30.8
*ea-1*	0	25.0	41.7	0	15.4
*eae*	100	0	0	0	12.8
*fimC*	100	100	100	100	100
*focG*	0	25.0	16.7	0	7.7
*gafD*	0	0	0	0	0
*hrA*	0	75.0	16.7	16.7	20.5
*iha*	0	0	0	0	0
*mat*	60.0	100	100	100	94.9
*nfaE*	0	0	0	0	0
*papAH*	0	75	0	0	7.7
*papC*	0	75	0	0	7.7
*papEF*	0	75	0	0	7.7
*papGII*,*III*	0	75	0	0	7.7
*sfa/foc*	0	100	16.7	0	15.4
*sfaS*	0	25	0	0	2.6
*tsh*	0	0	0	0	0
**Iron acquisition**					
*chuA*	100	100	100	66.7	84.6
*eitA/C*	0	0	0	0	0
*etsB*	0	0	0	0	0
*etsC*	0	0	8.3	0	2.6
*feoB*	100	100	100	100	100
*fyuA*	0	100	83.3	22.2	46.2
*ireA*	0	0	0	0	0
*iroN*	0	100	33.3	22.2	30.8
*irp2*	0	100	75.0	22.2	43.6
*iucD*	0	0	0	0	0
*iutA*	0	0	0	0	0
*sitA*	20	100	58.3	16.7	38.5
*sitD chrom*.	0	100	25.0	0	17.9
*sitD epis*.	0	0	0	0	0
**Protectins**					
*iss*	0	0	8.3	5.6	5.1
*kpsMTII*	0	75.0	41.7	5.6	23.1
*neuC*	0	0	0	0	0
*ompA*	100	100	100	100	100
*traT*	0	25.0	33.3	22.2	23.1
**Toxins**					
*EAST-1*	60.0	25.0	25.0	5.6	20.5
*cnf1/2*	0	0	0	0	0
*hlyA*	0	75.0	0	0	7.7
*hlyC*	0	75.0	0	0	7.7
*hlyF*	0	0	0	0	0
*sat*	0	0	0	0	0
*stx1*,*2*	0	0	0	0	0
*vat*	0	100	50.0	0	25.6
**Invasion-related**					
*ibeA*	60.0	50.0	41.7	0	25.6
*gimB*	0	0	0	0	0
*tia*	0	0	16.7	0	5.1
**Miscellaneaous**					
*cvaC*, *cvi/cva*	0	0	0	0	0
*escV*	100	0	0	0	12.8
*malX*	20.0	75.0	91.7	0	38.5
*ompT*	0	0	0	0	0
*pic*	0	50.0	16.7	0	10.3
*pks*	0	25.0	0	0	2.6
*puvA*	0	0	0	0	0

*E*. *albertii* strain IMT25440 was positive for the genes *eae*, *escV*, *fimC*, *ibeA*, *chuA*, *feoB*, and *ompA*

The 12 ExPEC-like isolates possessed 9 to 16 (median 12) VAGs, while the commensals harboured between 5 and 8 (median 7) VAGs.

P-fimbrial (*pap* operon), hemolysin A (*hlyA*, *hlyC*) and polyketide synthetase (*pks*) genes, all of which are highly linked with extraintestinal diseases, were exclusively present in ExPEC isolates. Likewise, the S-fimbrial (*sfa/foc*, *sfaS*, *focG*), ExPEC adhesin-I (*ea-I*), and vacuolating autotransporter (*vat*) genes were only detected in ExPEC and ExPEC-like isolates in different proportions (Table 2). None of our strains harboured the genes encoding for cytotoxic necrotizing factor *cnf* or secreted autotransporter toxin *sat*, which are commonly found among uropathogenic *E*. *coli* (UPEC). A high number of *E*. *coli* isolates (84.6%) possessed the hemin receptor gene *chuA*, which is not only involved in iron acquisition but is also a surrogate marker for the grouping of isolates into phylogenetic groups D or B2 [61]. Additionally, other iron acquisition genes were detected quite frequently among our sample material. Besides *chuA* and *feoB*, 33.3% of the *E*. *coli* isolates possessed 3–5 additional genes involved in bacterial capture of iron from the host.

A high proportion of isolates classified as commensal also carried the salmochelin receptor gene *chuA* (66.7%) while possessing less frequently other iron acquisition genes such as *fyuA* and *irp2* (22.2%), *iroN* (33.3%) as well as *sitA* (16.7%).

### MLST and phylogenetic diversity

The 39 *E*. *coli* isolates were assigned to 37 different STs, demonstrating a high diversity. Fifteen strains belonged to previously reported STs such as ST69, ST101, ST127, ST131 or ST372, which are regularly observed in humans and animals. Among these ST69 and ST131 represent typical ESBL-producing strains.

In addition, several new STs (n = 22) were assigned, which are highlighted in Fig 2. eBURST analysis including all available *E*. *coli* STs (n = 4909) provided in the MLST database and the STs identified in this study showed that all novel STs appeared as singletons instead of belonging to a ST complex representing a group of related strains.

In four animals, we could identify *E*. *coli* isolates with different STs and pathovar affiliation. One *M*. *torquata* harboured *E*. *coli* of three different STs (ST69, ST681, ST2802) and one *E*. *albertii* (ST3227), showing virulence gene profiles indicative of commensals, ExPEC-like and aEPEC in different organs, respectively. ST372 (classified as ExPEC) and ST131 (classified as ExPEC-like) were both isolated from the gut of one *E*. *helvum*. One animal (*M*. *torquata)* was co-colonized with an EPEC strain (ST722) and an aEPEC strain (ST3225), isolated from intestine and liver, respectively.

Diversity of STs was generally high, only few STs were present in more than one animal. In detail, ST1146 was found in three individuals of two different species (*M*. *torquata*, *E*. *helvum*), both originating from IFO. PFGE revealed that all ST1146 isolates were clones (S2 Fig). The same applied for ST3225, which occurred in two bats of the species *M*. *torquata* from different regions of the RC (IFO and PNOK). Both isolates were defined as EPEC and revealed identical virulence gene profiles as well as macrorestriction patterns, demonstrating their clonal nature.

The majority of strains were assigned to phylogroup B2 (46.2%), followed by group D (15.4%), ABD (23.1%), AxB1 (10.3%), B1 (2.6%) and A (2.6%).

Isolates belonging to group B2 harboured the highest number of VAGs (median 10.5), followed by group ABD (8.0), AxB1 (8.0), D (6.5), A and B1 (5.0).

Whole genome sequence analysis of the ST131 strain revealed that it is part of cluster B and shares with the other strains of this clade the *gyrA* and *parC* variants that are consistent with the strain´s susceptibility towards fluoroquinolones. Generally, this clade is not associated with AMR (unlike clade C) [50] (S3 Fig).

### Antimicrobial resistance

*E*. *coli* strains (n = 39) were susceptible to most of the tested antimicrobial substances according to clinical breakpoints given in the CLSI [44,45]. All isolates were susceptible to amikacin, gentamicin, amoxicillin/clavulanic acid, ampicillin, cefazolin, chloramphenicol, enrofloxacin, marbofloxacin, sulfamethoxazole/trimethoprime and tetracycline. Thirty-five strains (89.7%) were of intermediate resistance to doxycycline. Towards the cephalosporines cefovecin and cefalexin 28.2% resp. 20.5% of *E*. *coli* isolates showed intermediate resistance. *E*. *albertii* was susceptible towards all tested antimicrobials, although specific breakpoints are not provided for this species. None of the 40 *Escherichia* spp. strains revealed an ESBL-phenotype.

## Discussion

### Bacterial isolation

*E*. *coli* isolates were recovered from frozen tissue samples from 30 of 50 (60%) African fruit bats. Comparison to other studies on *E*. *coli* prevalence in fruit bats is difficult since most studies were performed on captive animals, which does not reflect the situation in the wild [16,36,62]. Other studies conducted on free-living fruit bats only include low sample sizes and are thus not representative for generalization [17,23,63,64].

More studies are available on insectivorous bats with *E*. *coli* prevalence of up to 28.5%, however differences in prevalence compared to fruit bats might be due to diet and environmental factors [12,13,17,18,65].

Animals living on the ground are generally more often exposed to *E*. *coli* since infection usually happens through oral-fecal transmission. Arboreal species, especially those that rarely touch the ground such as fruit bats, are thus less likely to come in contact with *E*. *coli*.

However, bats often share their habitat and food (fruits) with other mammal (e. g. monkeys) or bird species and could therefore get infected via these intermediate hosts. For instance, migratory birds have been suggested to contribute to the transmission of antibiotic resistant *E*. *coli* to wildlife in remote areas, where resistant strains of *E*. *coli* were found despite the absence of antibiotic exposure [66,67].

### Virulence gene typing and *E*. *coli* pathotype grouping

The present study provides evidence for fruit bats carrying potentially pathogenic *E*. *coli* pathovars, which are frequently involved in enteropathogenic and extraintestinal diseases in humans. Several strains showed typical VAG patterns of *E*. *coli* associated with extraintestinal diseases, however VAGs related to EPEC were found less frequently. Shiga-toxin genes *stx1* and *stx2*, which would be predictive for the presence STEC, were not present in any of the 40 tested *Escherichia* spp. isolates, which is in agreement with previous reports [17–19,65]. These studies together with our results indicate that bats may not be a reservoir of STEC and EHEC.

With EPEC, atypical EPEC and also one *eae*-positive *E*. *albertii*, we identified other enteropathogenic *E*. *coli* of potential relevance for human health. EPEC, one of the pathovars which causes diarrhoea, is a leading cause of childhood illness and death in developing countries [68], and also atypical EPEC are nowadays considered to be important in endemic diarrhoea in infants [69].

Our data are the first to provide evidence for the presence of typical EPEC and aEPEC in bats. This supports the work of Cabal et al. (2015), who suggested that aEPEC can be carried by bats, as they detected the EPEC-related genes *bfpA* and *eae* by real-time PCR in DNA from bats collected in Brazil [19]. Typical EPEC strains are rarely reported from domestic and wild animals [70–72], and humans are the major reservoir of these pathogens [27,73].

Another interesting aspect is the finding of an *eae*-positive *E*. *albertii* strain from the lung of a *M*. *torquata* bat sampled in PNOK. The clinical significance of *E*. *albertii* possessing intimin has yet not been fully elucidated, which may partly be due to the difficulty in discriminating *E*. *albertii* from other Enterobacteriaceae spp. by using routine bacterial identification systems based on biochemical properties [74].

Apart from EPEC and aEPEC, we also identified strains representing ExPEC or ExPEC-like pathogens. Due to the genetic and clinical complexity of ExPEC and also of different pathovars comprised in this group, such as uropathogenic *E*. *coli* (UPEC), avian pathogenic *E*. *coli* (APEC), strains associated with bloodstream infection in humans and mammalian hosts (septicemia-associated *E*. *coli* [SePEC]) or with meningitis in infants (new-born meningitis-associated *E*. *coli* [NMEC]), there is no clear definition of ExPEC based on single virulence genes as is the case for several intestinal pathogenic *E*. *coli* pathovars. Instead, there are multiple and redundant pathogenic mechanisms in this group of opportunistic pathogens. Although probably oversimplifying this complexity, we used a previously suggested molecular definition of ExPEC [53] as an initial basis to identify ExPEC among our bacterial samples. This identified four ExPEC strains from three individuals. Further investigation including the genes *ea-I* [59], *ibeA* [60], *pks*, *vat* and *sat* [54,55] as well as *hlyA*, *cnf* [57] revealed 12 ExPEC-like strains from 11 bats based on the presence of at least five ExPEC-related genes as described in the material and methods section. The pathogenic potential of the isolated strains has not been evaluated in this study, thus a final statement about pathogenicity cannot be made. In the same way, virulence cannot be completely ruled out for strains classified as commensals.

### Phylogenetic typing

It is known that B2 strains carry more VAGs compared to other phylogenetic groups [75]. Pathogenicity of *E*. *coli* has been linked to the possession of certain VAGs as well as to their phylogenetic background [76]. Phylogenetic analyses revealed that most of the *E*. *coli* strains can be categorized into four main groups, A, B1, B2 and D [77], as well as into the hybrid groups ABD and AxB1, deriving from the main groups [41]. Groups B2 and, at a lesser extent D, possess numerous specific VAGs, which are largely absent in non-pathogenic commensal strains [75,78]. B2 strains are also known to include most of the human and animal virulent ExPEC strains [79]. A substantial number of strains could be assigned to phylogenetic group B2, which is known to comprise most of the virulent ExPEC and a high proportion of EPEC strains.

Information about the distribution of phylogenetic groups of *E*. *coli* in bats is very limited. In contrast to our results, the majority of *E*. *coli* isolates from wild bats captured in Australia belonged to phylogenetic group B1 [13]. However, phylogenetic groups were equally distributed among the isolated *E*. *coli* in a more recent study on wild animals from Brazil that included bats [80]. Insofar, animals of the present study seem to harbour a remarkably high percentage of B2- *E*. *coli* isolates. Due to the multitude of VAGs, strains of phylogenetic group B2 seemed to have an advantage in the colonization of the intestinal tract, which may explain the high prevalence of B2 in the bats analysed.

B2 strains of the present study harboured the highest median of VAGs (10.5), followed by groups ABD (8.0), AxB1 (8.0), D (6.5), A and B1 (5.0). The majority of VAGs identified belonged to the groups of adhesins and iron acquisition systems, which might result from the anatomical conditions in the bat intestinal tract. Compared to other mammals of similar size, the intestinal tract of bats is three times shorter, which can result in reduced gastrointestinal passage times that is especially true in fruit bats [15]. To successfully adhere to the intestinal epithelial cells, *E*. *coli* requires specific adhesive factors. Furthermore, as *E*. *coli* have a very short time frame to supply itself with essential nutrients, it appears to require additional iron acquisition systems as were obviously present in the identified strains of this study.

Future studies should be conducted to see if there is really an association between adhesion of *E*. *coli* and gut passage time.

### MLST and macrorestriction analyses

In our study, we isolated 37 STs from wild fruit bats. Among those 22 STs were yet unknown in the MLST database. The finding of this high number of novel STs can easily be explained by the widely unexplored diversity of *E*. *coli* in African tropical wildlife.

Further on the results of the MLST analyses support the potentially zoonotic character of the isolated strains, as 15 STs have already been found among *E*. *coli* isolated from partly severe extraintestinal diseases in humans and/or animals (ST69, ST101, ST127, ST131, ST372, ST555, ST657, ST681, ST722, ST937, ST1146, ST1408, ST1597, ST1938 and ST2271).

Amongst those are some of special relevance, such as ST131, which is a very successful sequence type, often associated with ESBL-production and which has rapidly spread worldwide during the past two decades [81]. Additionally, ST69, ST101 and ST127 also predominantly consist of human ExPEC strains and have been associated with severe disease [82–85]. However, the virulence features of the isolated strains were much less pronounced, indicating these particular strains of STs as being less virulent compared to their counterparts circulating in the human population. Only a few strains with a suggested virulence (according to VAGs and STs) were identified, which would still have to be verified in pathogenicity studies. Also future in depth comparative whole genome sequence analyses are needed to provide insight into the microevolution of this interesting strain collection.

Nevertheless, the risk of transmission of these STs to humans or domestic animals still remains. Considering that fruit bats, including the species investigated here, are frequently hunted as bushmeat, there exists ample opportunities for direct pathogen transmission, e.g. through preparation and consumption. In addition, several fruit bat species have adapted to human environments where they can exist in large populations and where the potential for contact is likely higher than in the current study through environmental contamination, including food markets. Like many other wild animals, bats therefore should also be considered as an additional source for *E*. *coli* with pathogenic potential.

PFGE-analyses revealed clonal relatedness of identical STs in both different bat species within the same area and different individuals of the same species, but collected in distant regions of the country. This clearly indicates intra-and interspecies transmission of *E*. *coli* and suggests that strains are widely distributed.

### Antimicrobial resistance

None of the isolated strains, including those typically associated with ESBL-production (ST69 and ST131) [81,82,86–88], showed any resistance towards the tested antimicrobial agents. The result is not surprising as mainly human proximity is suggested to be responsible for presence and increase of antibiotic resistances in animals [13,33,89,90]. Bats of this study all derived from areas with very low human population density and thus a low likelihood of human to bat transmission of *E*. *coli*. This indicates that known human strains appear to be more frequently found in wild animals as originally assumed as part of the naïve *E*. *coli* population. Interestingly, only very few of the STs isolated showed intermediate resistance to certain antimicrobial substances tested. This, together with the predominantly arboreal lifestyle of the fruit bats of the present study, points towards a rather “naïve” population of *E*. *coli* in the fruit bats studied here. Even the presence of a ST131 strain fits into this picture. This phylogenetic lineage of *E*.*coli* has received a lot of attention in recent years; on the one hand due to its worldwide success in circulating in different hosts, on the other hand due to its association with ESBL resistance. Whole genome analysis revealed that this particular ST131 strain rather belongs to an evolutionary older clade within the ST131 phylogeny that is usually not associated with antibiotic resistance. The high number of novel STs also reflects a more naïve population. However, further studies including whole genome sequencing and evolutionary analyses should be performed to describe the *E*. *coli* diversity circulating in wildlife—particularly in the understudied tropical setting.

## Supporting information

S1 Fig Phylogenetic tree of strains isolated from African fruit bats. (PDF)

S2 Fig PFGE of selected strains/ clones. (PDF)

S3 Fig Phylogenetic tree of ST131 isolated from African fruit bat. (PDF)

S1 Table Primers used for virulence gene detection. (PDF)

S2 Table Categorization schemes of *E*. *coli* isolates, based on the presence of VAGs. (PDF)
