# Supplementary material for: Highly diverse and antimicrobial susceptible Escherichia coli display a naïve bacterial population in fruit bats from the Republic of Congo
Source: PLoS One. 2017 Jul 12;12(7):e0178146. doi: 10.1371/journal.pone.0178146 (PMC5507484; doi:10.1371/journal.pone.0178146)
Supplement: S1 Fig — (PDF) [file pone.0178146.s001.pdf]

S1 Fig. Phylogenetic tree of strains isolated from African fruit bats

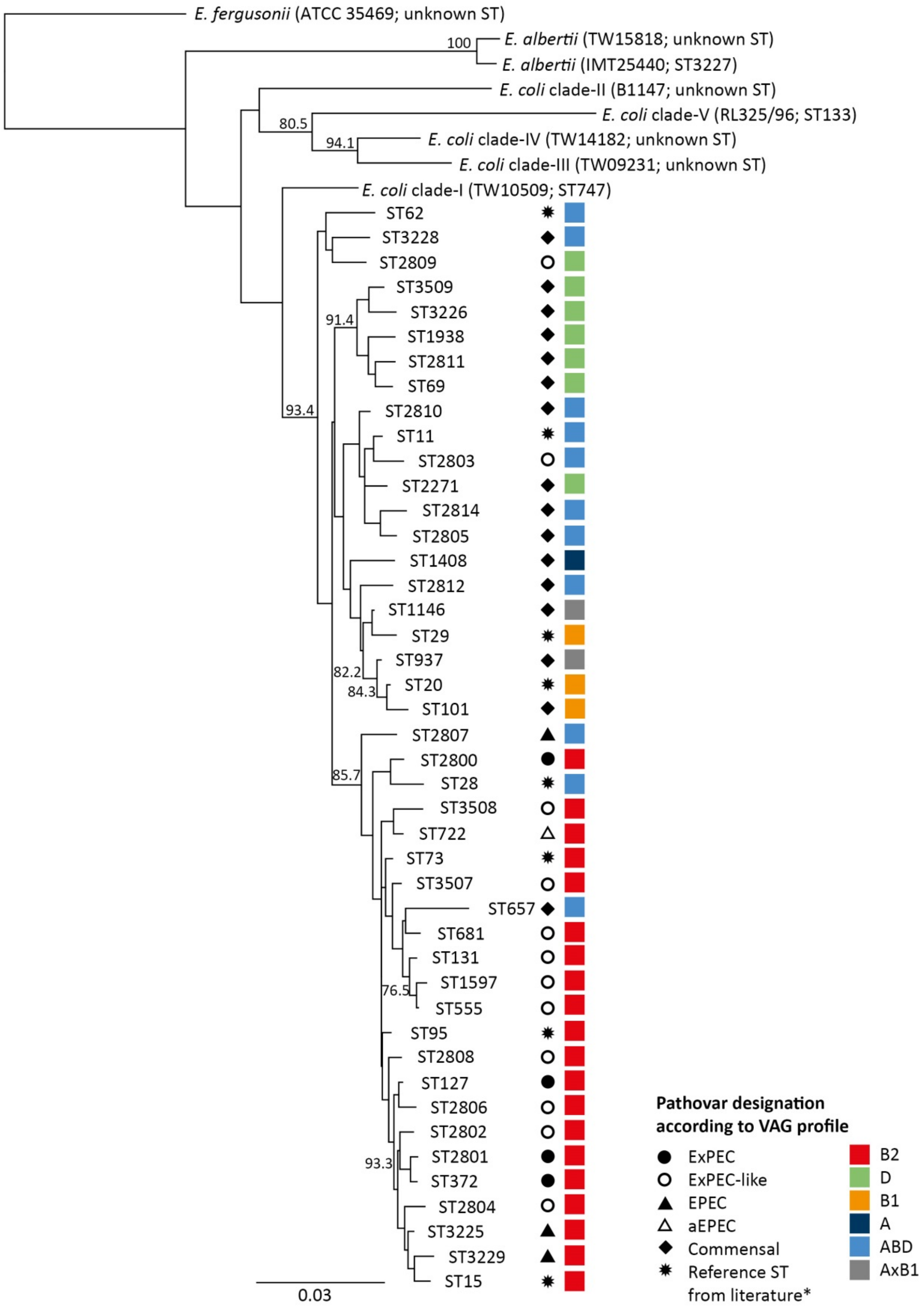

Phylogenetic tree based on concatenated sequences (3423 bp) of seven housekeeping genes used for MLST analysis showing the genetic relationship of 39 *E. coli* and one *E. albertii* isolate from bats and virulence-gene-based assignment to pathovars. Sequence types

(reference STs) frequently associated with ExPEC (ST62, ST95, ST73), EPEC or aEPEC (ST15, ST20, ST28, ST29), and commensals (ST10) (http://mlst.warwick.ac.uk/mlst/dbs/Ecoli; accessed 10.02.2016) are included for comparative purposes. Likewise, members of the “second population” *Escherichia* clades I-V, of *E. albertii* and *E. fergusonii* are also included (45,90,91). The maximum likelihood tree was created with PhyML (substitution model HKY85) after alignment of sequences with MAFFT v7.017, both implemented in Geneious. Numbers at nodes represent bootstrap values >70%, expressed as percentages of 1000 replications. Bar: 0.03 substitutions per sequence position.
