# Supplementary material for: Highly diverse and antimicrobial susceptible Escherichia coli display a naïve bacterial population in fruit bats from the Republic of Congo
Source: PLoS One. 2017 Jul 12;12(7):e0178146. doi: 10.1371/journal.pone.0178146 (PMC5507484; doi:10.1371/journal.pone.0178146)
Supplement: S2 Fig — (PDF) [file pone.0178146.s002.pdf]

S2 Fig. PFGE of selected strains / clones

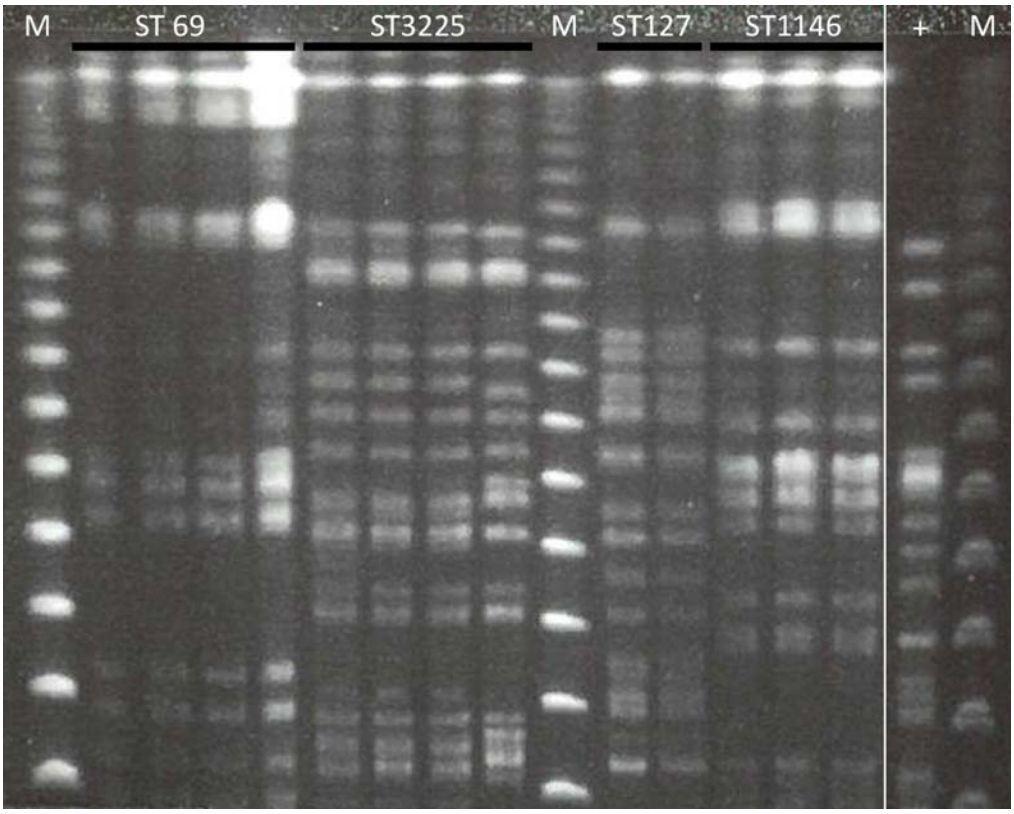

CHEF-PFGE-electropherogram of XbaI-restricted genomic DNA selected strains (angle, 1208; voltage 6 V/cm; pulsed-field times 5–12 s for the first 11 h and 20–50 s for the next 11 h; ramping, linear). M: lambda ladder PFG marker; + = positive control
