# Supplementary material for: Highly diverse and antimicrobial susceptible Escherichia coli display a naïve bacterial population in fruit bats from the Republic of Congo
Source: PLoS One. 2017 Jul 12;12(7):e0178146. doi: 10.1371/journal.pone.0178146 (PMC5507484; doi:10.1371/journal.pone.0178146)
Supplement: S3 Fig — (PDF) [file pone.0178146.s003.pdf]

S3 Fig. Phylogenetic tree of ST131

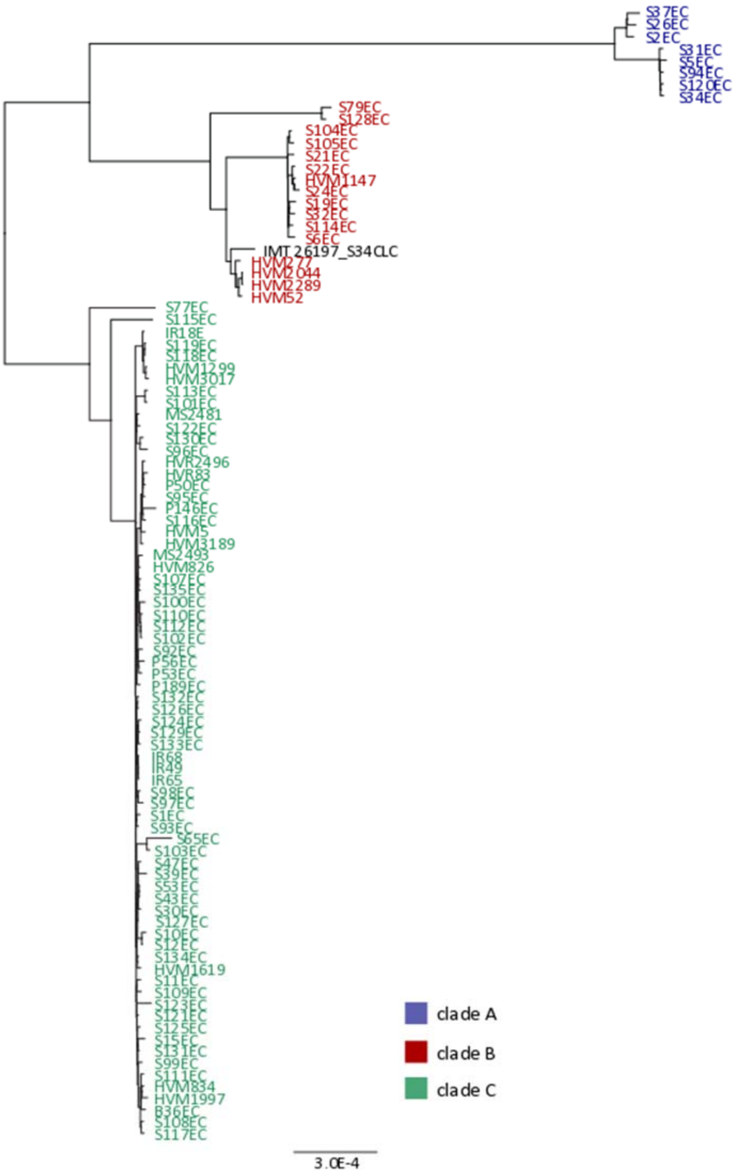

Whole genome phylogeny on the basis of the maximum common genome (MCG) (92) of our ST131 strain and public available ST131 strains from a comparative study (50). Allelic sequences of 3698 orthologous genes where aligned and a maximum likelihood phylogeny was calculated with RAxML 8.1.
