# Supplementary material for: Highly diverse and antimicrobial susceptible Escherichia coli display a naïve bacterial population in fruit bats from the Republic of Congo
Source: PLoS One. 2017 Jul 12;12(7):e0178146. doi: 10.1371/journal.pone.0178146 (PMC5507484; doi:10.1371/journal.pone.0178146)
Supplement: S1 Table — (PDF) [file pone.0178146.s004.pdf]

**S1 Table. Primers used for virulence gene identification;** MP=Multiplex PCR

| Abbreviation/ gene | Description | Primer no. | Product (bp) | Sequence (5'-3', sense (s), antisense (as)) | Reference |
|---|---|---|---|---|---|
| **MP I** | | | | | |
| *afa/draBC* | afimbrial, Dr specific adhesin | 1604/1605 | 810 bp | s: TAAGGAAGTGAAGGAGCGTG/ as: CCAGTAACTGTCCGTGACA | (1) |
| *fimC* | Typ 1 fimbria | 33/34 | 477 bp | s: GGGTAGAAAATGCCGATGGTG/ as: CGTCATTTTGGGGGTAAGTGC | (1) |
| *hrA/hek* | heat resistant hemagglutinin | 1546/1547 | 537 bp | s: GTAACTCACACTGCTGTCACCT/ as: TCACTTGCAGACCAGCGTTTC | (1) |
| *sfa/foc* | s-fimbriae adhesin/ F1 fimbriae | 1566/1567 | 1242 bp | s: CGGAGAACTGGGTGCATCTTA/ as: GTCCTGACTCATCTGAAACTGCA | (1) |
| *hlyA* | hemolysin A | 21/22 | 352 bp | s: GTCCATTGCCGATAAGTTT/ as: AAGTAATTTTTGCCGTGTTTT | (2) |
| *pic* | serin protease autotransporter | 1071/1072 | 412 bp | s: ACTGGA TCT TAA GGC TCA GG/ as: TGGAATATCAGGGTGCCACT | (1) |
| *kpsMTII* | group II capsule antigen | 1544/2084 | 270 bp | s: CATCCAGACGATAAGCATGAGCA/ as: GCGCATTTGCTGATACTGTTG | (3) |
| *neuC* | K1 capsular polysaccharide | 1904/1905 | 676 bp | s: GGTGGTACATTCCGGGATGTC/ as: AGGTGAAAAGCCTGGTAGTGTG | (4) |
| *RPai (malX)* | pathogenicity associated island marker CFT 073 | 1610/1611 | 922 bp | s: GGACATCCTGTTACAGCGCGCA/ as: TCGCCACCAATCACAGCCGAAC | (3) |
| **MP II** | | | | | |
| *chuA* | *E. coli* haem utilization | 445/446 | 278 bp | s: GACGAACCAACGGTCAGGAT/ as: TGCCGCCAGTACCAAAGACA | (5) |
| *gimB* | genetic island associated with meningitis | 1261/1262 | 736 bp | s: TCCAGATTGAGCATATCCC/ as: CCTGTAACATGTTGGCTTCA | (1) |
| *ibeA* | invasion of brain endothelium | 1056/1764 | 342 bp | s: TGGAACCCGCTCGTAATATAC/ as: CTGCCTGTTCAAGCATTGCA | (1) |
| *iroN* | iron receptor | 1608/1609 | 847 bp | s: ATCCTCTGGTCGCTAACTG/ as: CTGCACTGGAAGAACTGTTCT | (1) |
| *ompA* | outer membrane protein | 904/905 | 919 bp | s: AGCTATCGCGATTGCAGTG/ as: GGTGTTGCCAGTAACCGG | (1) |
| *traT* | transfer protein | 902/903 | 430 bp | s: GTGGTGCGATGAGCACAG/ as: TAGTTCACATCTTCCACCATCG | (1) |
| *sitD* (chrom.) | salmonella iron transport system gene | 1765/1766 | 554 bp | s: ACTCCCATACACAGGATCTG/ as: CTGTCTGTGTCCGGAATGA | (1) |
| *sitD* (episomal) | salmonella iron transport system gene | 1762/1763 | 1052 bp | s: TTGAGAACGACAGCGACTTC/ as: CTATCGAGCAGGTGAGGA | (1) |

| | | | | | |
|---|---|---|---|---|---|
| **MP III** | | | | | |
| *astA (East-1)* | heat stable cytotoxin associated with enteroaggregative *E. coli* | 7/8 u. 762/763 | 116 bp | s: TGCCATCAACACAGTATATCC/ as: TAGGATCCTCAGGTCGCGAGTGACGGC | (6) |
| *colV (cvi/cva)* | structural genes of colicin V operon | 1828/1829 | 598 bp | s: TCCAAGCGGACCCCTTATAG/ as: CGCAGCATAGTTCCATGCT | (1) |
| *irp2* | iron repressible protein | 15/1066 | 413 bp | s: AAGGATTCGCTGTTACCGGAC/ as: AACTCCTGATACAGGTGGC | (7) |
| *iss* | increased serum survival | 1847/1848 | 309 bp | s: ATCACATAGGATTCTGCCG/ as: CAGCGGAGTATAGATGCCA | (1) |
| *iucD* | yersinia bactin | 23/24 | 714 bp | s: ACAAAAAGTTCTATCGCTTCC/ as: CCTGATCCAGATGATGCTC | (8) |
| *papC* | pilus associated with pyelonephritis | 31/32 | 501 bp | s: TGATATCACGCAGTCAGTAGC/ as: CCGGCCATATTCACATAAC | (8) |
| *tsh* | temperature sensitive hemagglutinin | 1028/1029 | 824 bp | s: ACTATTCTCTGCAGGAAGTC/ as: CTTCCGATGTTCTGAACGT | (1) |
| *vat* | vacuolating autotransporter toxin | 1069/1070 | 981 bp | s: TCCTGGGACATAATGGTCAG/ as: GTGTCAGAACGGAATTGTC | (2) |
| **MP IV** | | | | | |
| *cnf1/2* | cytotoxic necrotizing factor | 11/12 | 446 bp | s: TCGTTATAAAATCAAACAGTG/ as: CTTTACAATATTGACATGCTG | (2) |
| *csgA* | "curlin subunit gene A" (Curli fimbria) | 1865/1866 | 250 bp | s: ACTCTGACTTGACTATTACC/ as: AGATGCAGTCTGGTCAAC | (9) |
| *feoB* | ferrous iron transport | 1664/1665 | 1095 bp | s: TGAGGACTCCGGCTATATGG/ as: ATAACTGCCAGAATGCACACC | (10) |
| *fyuA* | ferric yersinia uptake (yersiniabactin receptor | 13/14 | 775 bp | s: GCGACGGGAAGCGATGACTTA/ as: CGCAGTAGGCACGATGTTGTA | (7) |
| *ireA* | iron responsive element | 1867/1868 | 384 bp | s: ATTGCCGTGATGTGTTCTGC/ as: CACGGATCACTTCAATGCGT | (1) |
| *mat* | meningitis associated and temperature regulated fimbriae | 2086/2087 | 899 bp | s: TATACGCTGGACTGAGTCGTG/ as: CAGGTAGCGTCGAACTGTA | (1) |
| *sat* | secretet autotransporter toxin | 840/841 | 667 bp | s: TGCTGGCTCTGGAGGAAC/ as: TTGAACATTCAGAGTACCGGG | (1) |
| *tia* | toxigenic invasion locus | 1941/1942 | 512 bp | s: AGCGCTTCCGTCAGGACTT/ as: ACCAGCATCCAGATAGCGAT | (1) |
| **MP V** | | | | | |
| *bfp* | bundle forming pili | 3087/3088 | 910 bp | s: GACACCTCATTGCTGAAGTCG/ as: CCAGAACACCTCCGTTATGC | (11) |
| *escv* | escherichia secretion complex | 3085/3086 | 544 bp | s: ATTCTGGCTCTCTTCTTCTTTATGGCTG/ as: CGTCCCCTTTTACAAACTTCATCGC | (12) |
| *stx1* | shigatoxin 1 | 3089/3090 | 244 bp | s: CGATGTTACGGTTTGTTACTGTGACAGC/ as: AATGCCACGCTTCCCAGAATTG | (12) |
| *stx2* | Shigatoxin 2 | 3091/3092 | 324 bp | s: GTTTTGACCATCTTCGTCTGATTATTGAG/ as: AGCGTAAGGCTTCTGCTGTGAC | (11) |

| | | | | | |
|---|---|---|---|---|---|
| **MP VI b** | | | | | |
| *focG* | fimbriae of serotype 1C | 3174/3175 | 360 bp | s: CAGCACAGGCAGTGGATACGA/ as: GAATGTCGCCTGCCCATTGCT | (3) |
| *gafD* | N-acetyl-D-Glucosaminspezifisches fimbrial lectin | 3178/3179 | 952 bp | s: TGTTGGACCGTCTCAGGGCTC/ as: CTCCCGGAACTCGCTGTTACT | (3) |
| *iutA* | iron uptake transport | 3182/3183 | 300 bp | s: GGCTGGACATCATGGGAACTGG/ as: CGTCGGGAACGGGTAGAATCG | (3) |
| *nfaE* | nonfimbrial adhesin | 3180/3181 | 559 bp | s:GCTTACTGATTCTGGGATGGA / as: CGGTGGCCGAGTCATATGCCA | (3) |
| *papAH* | pilus associated with pyelonephritis | 3157/3158 | 721 bp | s: ATGGCAGTGGTGTCTTTTGGTG/ as: CGTCCCACCATACGTGCTCTTC | (3) |
| **MP VI c** | | | | | |
| *bmaE* | blood group M-specific adhesin | 3176/3177 | 505 bp | s: ATGGCGCTAACTTGCCATGCTG/ as: AGGGGGACATATAGCCCCCTTC | (3) |
| *hlyC* | hemolysin C | 4199/4200 | 556 bp | s: AGGTTCTTGGGCATGTATCCT/ as: TTGCTTTGCAGACTGCAGTGT | (13) |
| *papEF* | pilus associated with pyelonephritis | 3159/3160 | 336 bp | s: GCAACAGCAACGCTGGTTGCATCAT/ as: AGAGAGAGCCACTCTTATACGGACA | (14) |
| *papGII, III* | pilus associated with pyelonephritis | 3161/3162 | 1057 bp | s: CTGTAATTACGGAAGTGATTTCTG/ as: ACTATCCGGCTCCGGATAAACCAT | (15) |
| *sfaS* | s-fimbriae adhesin | 3172/3173 | 242 bp | s: GTGGATACGACGATTACTGTG/ as: CCGCCAGCATTCCCTGTATTC | (3) |
| **MP VII** | | | | | |
| *hlyF* | hemolysin F | 3818/3819 | 250 bp | s: ATGGATCCTCGTCTTGATG/ as: GCTCATTGCCAGTATGTCA | this work |
| *ompT* | outer membrane protein | 3820/3821 | 610 bp | s: TTGCTACTGCACTCTCAGC/ as: CGACAGATACTCTGGGTAACA | this work |
| *pks* | polycetide synthase | 2540/2541 | 300 bp | s: CGCTTCATCAACACGCTTTA/ as: CCATCGCCTATCACCTCAAC | H. Karch |
| **MP VIII** | | | | | |
| *iha* | iron regulated gene-homologe adhesin | 1943/1944 | 2088 bp | s: ATGCGAATAACCATTTTGGCTTCC/ as: TCAGAACTGATAGTTCAGCGACA | this work |
| *puvA* | previously unidentified virulence gene | 1602/1603 | 521 bp | s: CACACTTGTATAGCAACCCGT/ as: TGCACTGTCAGATACAGGTG | this work |
| *ea/ I* | novel adhesin | 2512/2559 | 761 bp | s: ATGCAATGGCAGTACCCTTC/ as: ATAAACACAATATGGCGCTCG | (16) |

| **MP IX** | | | | | |
|---|---|---|---|---|---|
| *cvaC* | colV-plasmid-structure protein | 4195/4196 | 679 bp | s: CACACACAAACGGGAGCTGTT/ as: CTTCCCGCAGCATAGTTCCAT | (3) |
| *eitA* | *E. coli* iron transport | 4185/4186 | 450 bp | s: ACGCCGGGTTAATAGTTGGGAGATAG/ as: ATCGATAGCGTCAGCCCGGAAGTTAG | (17) |
| *etsA* | *E. coli* transport system | 4189/4190 | 284 bp | s: CAACTGGGCGGGAACGAAATCAGGA/ as: TCAGTTCCGCGCTGGCAACAACCTAC | (17) |
| *etsB* | *E. coli* iron transport | 4191/4192 | 380 bp | s: CAGCAGCGCTTCGGACAAAATCTCCT/ as: TTCCCCACCACTCTCCGTTCTCAAAC | (17) |
| *eitB* | *E. coli* transport system | 4187/4188 | 537 bp | s: TGATGCCCCGCCAAACTCAAGA/ as: ATGCGCCGGCCTGACATAAGTGCTAA | (17) |
| *sitA* | *Salmonella* iron transport system gene | 4193/4194 | 608 bp | s: AGGGGGCACAACTGATTCTCG/ as: TACCGGGCCGTTTTCTGTGC | (18) |


1. Ewers C, Li G, Wilking H, Kiessling S, Alt K, Antáo E-M, et al. Avian pathogenic, uropathogenic, and newborn meningitis-causing *Escherichia coli*: how closely related are they? Int J Med Microbiol. 2007 Jun;297(3):163–76.
2. Ewers C, Janssen T, Kiessling S, Philipp HC, Wieler LH. Molecular epidemiology of avian pathogenic *Escherichia coli* (APEC) isolated from colisepticemia in poultry. Vet Microbiol. 2004 Nov 30;104(1-2):91–101.
3. Johnson J, Stell A. Extended virulence genotypes of *Escherichia coli* strains from patients with urosepsis in relation to phylogeny and host compromise. J Infect Dis. 2000;181(January):53–9.
4. Zapata G, Crowley JM, Vann WF. Sequence and expression of the *Escherichia coli* K1 neuC gene product. Journal of Bacteriology. 1992. p. 315–9.
5. Clermont O, Bonacorsi S, Bingen E, Bonacorsi P. Rapid and Simple Determination of the *Escherichia coli* Phylogenetic Group. Appl Environ Microbiol. 2000;66(10):4555–8.

6. Yamamoto T, Echeverria P. Detection of the enteroaggregative *Escherichia coli* heat-stable enterotoxin 1 gene sequences in enterotoxigenic *E. coli* strains pathogenic for humans. Infect Immun. 1996;64(4):1441–5.

7. Schubert S, Rakin A, Karch H, Carniel E, Heesemann J. Prevalence of the “high-pathogenicity island” of *Yersinia* species among *Escherichia coli* strains that are pathogenic to humans. Infect Immun. 1998;66(2):480–5.

8. Janssen T, Schwarz C, Preikschat P, Voss M, Philipp HC, Wieler LH. Virulence-associated genes in avian pathogenic *Escherichia coli* (APEC) isolated from internal organs of poultry having died from colibacillosis. Int J Med Microbiol. 2001;291:371–8.

9. Maurer JJ, Brown TP, Steffens WL, Thayer SG. The occurrence of ambient temperature-regulated adhesins, curli, and the temperature-sensitive hemagglutinin tsh among avian *Escherichia coli*. Avian Dis. 1998;42(1):106–18.

10. Römer A. Vergleichende molekulare, epidemiologische und phylogenetische Untersuchungen zur Anpassung von *Escherichia coli* bei Wild- und Hausschweinen. Freie Universität, Berlin; 2009.

11. Müller D, Greune L, Heusipp G, Karch H, Fruth A, Tschäpe H, et al. Identification of unconventional intestinal pathogenic *Escherichia coli* isolates expressing intermediate virulence factor profiles by using a novel single-step multiplex PCR. Appl Environ Microbiol. 2007 May;73(10):3380–90.

12. Müller D, Hagedorn P, Brast S, Heusipp G, Bielaszewska M, Friedrich AW, et al. Rapid identification and differentiation of clinical isolates of enteropathogenic *Escherichia coli* (EPEC), atypical EPEC, and Shiga toxin-producing *Escherichia coli* by a one-step multiplex PCR method. J Clin Microbiol. 2006 Jul;44(7):2626–9.

13. Bingen E, Picard B, Brahimi N, Desjardins P, Elion J, Denamur E. Phylogenetic Analysis of *Escherichia coli* Strains Causing Neonatal Meningitis Suggests Horizontal Gene Transfer from a Predominant Pool of Highly Virulent B2 Group Strains. J Infect Dis. 1998;177:642–50.

14. Yamamoto S, Terai A, Yuri K, Kurazono H, Takeda Y, Yoshida O. Detection of urovirulence factors in *Escherichia coli* by multiplex polymerase chain reaction. FEMS Immunol Med Microbiol. 1995;12(2):85–90.

15. Marklund BI, Tennent JM, Garcia E, Hamers A, Baga M, Lindberg F, et al. Horizontal gene transfer of the *Escherichia coli* pap and prs pili operons as a mechanism for the development of tissue-specific adhesive properties. Mol Microbiol. 1992;6(16):2225–42.

16. Antão EM, Ewers C, Gürlebeck D, Preisinger R, Homeier T, Li G, et al. Signature-tagged mutagenesis in a chicken infection model leads to the identification of a novel avian pathogenic *Escherichia coli* fimbrial adhesin. PLoS One. 2009;4(11).

17. Johnson TJ, Siek KE, Johnson SJ, Nolan LK. DNA Sequence of a ColV Plasmid and Prevalence of Selected Plasmid-Encoded Virulence Genes among Avian *Escherichia coli* Strains. J Bacteriol. 2006;188(2):745–58.

18. Rodriguez-Siek KE, Giddingsb CW, Doetkottc C, Johnson TJ, Nolan LK. Characterizing the APEC pathotype. VetRes. 2005;36:241–56.
