# Supplementary material for: Highly diverse and antimicrobial susceptible Escherichia coli display a naïve bacterial population in fruit bats from the Republic of Congo
Source: PLoS One. 2017 Jul 12;12(7):e0178146. doi: 10.1371/journal.pone.0178146 (PMC5507484; doi:10.1371/journal.pone.0178146)
Supplement: S2 Table — (PDF) [file pone.0178146.s005.pdf]

S2 Table. Categorization schemes of *E. coli* isolates, based on the presence of VAGs

	number of VAGs	gene	Pathovar	Ref
Scheme 1	≥2	*iutA, kpsMT II, papA/C, sfa/foc, afa/dra*	ExPEC	(52)
Scheme 2	≥5	*kpsMT II, neuC, ea-I, hrA, sfa/foc, tsh, cnf, hlyA/C/F, vat, sat, ibeA, chuA, sitA/D, etsB/C, eitA/C, fyuA, ireA, iroN, irp2, iucD, malX, cvi/cvaC, iss, ompT, pks*	ExPEC-like	this work
